# Correlates of somatic symptom disorder among internally displaced persons in Ogoja displacement settlements, Nigeria: a cross-sectional study

**DOI:** 10.4314/ahs.v23i3.81

**Published:** 2023-09

**Authors:** Ugbe Maurice-Joel Ugbe, Ekpereonne Babatunde Esu, Obiageli Chiezey Onwusaka, Marvin Muji Bisongedam, Elizabeth Libuo-Beshel Nji, Joseph Ajah Efut, Ofem Irom Ekpo, Faith Ubi Okoi

**Affiliations:** Department of Public Health, University of Calabar, Calabar, Nigeria

**Keywords:** Somatoform, somatic symptom disorder, internally displaced adults

## Abstract

**Background:**

Experiences of displacement have been associated with the prevalence of mental health disorders owing to certain factors.

**Objectives:**

This study aimed to identify the correlates of Somatic Symptom Disorder (SSD) among internally displaced adults in Ogoja displacement settlements, Nigeria.

**Methods:**

This was a cross-sectional study of 335 respondents. SSD was assessed using the SOM-SCL section of the Common Mental Disorder Questionnaire while a semi-structured questionnaire was used to collect data on sociodemographic and displacement-related factors. Data were analysed using descriptive statistics, Chi-square, and multivariable logistic regression.

**Result:**

The prevalence of somatoform disorder was 59.1%. Factors found to be significant in each bivariate Chi-square analysis were modelled for the mental disorder. The multivariate analysis revealed that being married (AOR=2.80; *p*=0.020) prolonged displacement (AOR=3.29; *p*=0.003), discrimination (AOR=2.25; *p*=0.010), disease outbreak (AOR=1.92; *p*=0.030), loss of loved ones (AOR=1.34; *p*=0.028), overcrowded households (AOR=2.30; *p*=0.008), and fear of reprisals (AOR=2.05; *p*=0.026) were significantly associated with somatoform disorder.

**Conclusion:**

The findings suggest that the high prevalence of the studied outcome is related to several stressors and events among Internally displaced persons. Evidence-based mental health support efforts by different bodies in creating and routinely arranging mental health clinical interventions for this population is recommended.

## Introduction

Human displacement remains one of the most significant humanitarian challenges facing the world.^1^ The global space has been plagued with rising new displacements annually due to crises and disasters. Despite novel frameworks from action in several countries, the primary concern with regards to the background of internal displacement are still sparsely being properly attended to.^2^

Drivers of prolonged displacement include political crises, severe poverty, and global warming as most IDs are making their way back to conflict-ridden areas without means of livelihoods. Globally, over 50.8 million persons have been displaced due to both conflicts or violence and disasters.^2^ Further perusal into new displacements indicates that 8.5 million persons and 24.9 million persons were displaced by conflicts/ violence and disasters, respectively. Approximately 6.4% of the global total for new displacements for 2019 was recorded displacements from the Americas, 0.3% in Europe and Central Asia, 24.1% in Sub-Saharan Africa, 9.6% in the Middle East and North Africa, 30% in South Asia, and 29.6% in East Asia and Pacific.^2^ In Africa, there is a higher concentration of displacement due to conflicts and wars. Nigeria, Ethiopia, the Democratic Republic of Congo, Cameroon, and Sudan show the existing problems of displacement as the years go by.^3^ There has been minimal will to subdue such situations from escalating, thus leading to the increased figures that are being presented each year.

In Nigeria, it is estimated that 20-30% of the population suffers from mental disorders.^4^ The World Health Organization's Assessment Instrument for Mental Health Systems (AIMS) 2006 highlighted the neglect of mental health issues in Nigeria. Besides, policy documents on psychological health were found to be outdated without further revisions to include situations of crises and violence that have plagued the country in present times.^4^

Losing home, loved ones, and being destabilized has its ripple effects and leads to many adverse mental health outcomes. Kronick 5 stated that the prevalence of Common Mental Disorders in resettled refugees, who at some point also attained IDP status range from 4-44%. The dangers of traumatic events cause fear, anxiety, and withdrawal.^6^ Conflicts are followed by living in conditions of uncertainty, fear, somatic problems, and anxiety, which continue to impact the displaced persons negatively. While depression, anxiety and Post Traumatic Stress Disorder (PTSD) are more commonly explored among war affected persons, there is still paucity of data regarding Somatic Symptom Disorder among the aforementioned population. Somatoform disorder is known to manifest as physical disorders in the absence of any known underlying medical problems, while producing symptoms such as unexplained pains in the chest, abdomen, or limbs as well as sleeplessness.^7^ Some of the risk factors associated with Somatoform disorder include exposure to traumatic conditions, comorbid mental conditions, and several socioeconomic deprivations, all of which are usually increased in conflict situations.^7^ Despite the effect of this mental condition, limited studies have emerged from low- and middle-income countries where most of the world's IDPs reside. Only studies 8, 9 have been able to magnify this situation in low resource settings.

Accordingly, this study aimed at examining the prevalence, socio-demographic correlates, and associated factors of somatoform disorder among internally displaced adults. Operationally, the study postulated two hypotheses to guide the study thus:

1. There is no statistically significant relationship between selected socio-demographic factors and diagnoses of somatoform disorder among internally displaced adults in the study area
2. There is no statistically significant relationship between displacement-related factors and diagnoses of somatoform disorder among internally displaced adults in the study area

## Methods

### Study Area, Scope and Design

Ogoja Local Government Area is located in Cross River State, Nigeria, lying 6o30′N 8o40′E, about 300km north of Calabar, the state capital.^10^ It has an area of 972km^2^ which is about 375 sq. mi and a population of 171,901 as of the 2006 population census.^11^ This study comprised adult IDPs aged 18 years and above who had been displaced by natural and man-made causes in Ogoja LGA. Data for this present study were collected from only indigenes of Nigeria who were displaced and now reside in the host communities in Ogoja.

This was a cross-sectional descriptive study employing a quantitative approach in determining the prevalence, sociodemographic correlates, and associated factors of SSD among displaced adults in the study area.

### Sample Procedure

The sample size for this study was determined using Fischer's formula for estimating sample size of Cross-sectional studies.


$$\begin{array}{c} \text{n}= \end{array}\begin{array}{c} \text{Z}{\text{α}}^{2}\text{pq}\\ {\text{d}}^{2} \end{array}$$

n= calculated sample size; Z = Standard normal variate- 1.96 (at 95% confidence interval); p= proportion of prevalence of any Common Mental Disorder (CMD) was 0.64 as reported in a study 12

q = proportion without the outcome (0.36); d= margin of error for proportion being estimated (0.05)


$$n=\frac{1.96^{2}\times 0.64\times 0.36}{0.05^{2}}=354$$

Having knowledge about the number of IDPs in the area to be 4,600 persons, we applied finite population correction and adjusted for a 10% non-response rate. This amounted to a calculated sample size of 366 respondents.

### Sampling Procedure

A multi-stage sampling technique using probability and non-probability methods was employed to recruit participants for the study. Due to the influx of IDPs in specific locations in the study setting, purposive sampling was utilized to select three wards majorly playing host to internally displaced persons in the study setting. Only wards that had a high IDP presence were selected for the study. Purposive sampling was used to select all ten communities of the three wards. Due to the hard-to-reach nature of IDPs and lack of reachable IDP settlements, snowball sampling technique was used in identifying households or settlements where IDPs resided. Only one consenting adult (aged 18 years and above) IDP in each household was recruited using simple random sampling in cases of multiple adults in one household.

### Instruments for data collection

A semi-structured questionnaire was used to collect demographic information of all respondents and displacement-related factors associated with somatoform disorder. The SCL-SOM section of the Common Mental Disorder Questionnaire (CMDQ) was used to diagnose this condition. Items 1-12 on the CMDQ depict symptoms of somatoform disorder. The CMDQ has proven to possess high diagnostic accuracy and exceptional external validity 13 and it is a 36-Item questionnaire that covers signs and symptoms generally linked with Somatic Symptom Disorders (SSDs), general anxiety, depression, and substance use and abuse. Its questions are constructed by adopting symptoms criteria from the SCL-90-R. [13] Each question is rated on a five-point Likert scale of distress with responses ranging from ‘not at all to ‘extremely’ (0-4).

### Method of data collection

Trained research assistants were recruited for the study. They were also recruited based on their knowledge of the local languages and navigation around the community. All the aforementioned instruments were interviewer-administered using Open Data Kit (ODK) collect software which was linked with Kobo tool box online database. A total of 335 displaced adults who gave their consents, were recruited, and were willing to participate.

### Method of data analysis

Data were extracted from ODK collect and downloaded into Microsoft Excel to scan for entry completeness. Data were imported into and analysed using Statistical Product and Service Provision -version 23. All responses in the CMDQ were dichotomized between ‘not at all’ and ‘a little’ as validated by Christensen, Fink 13. This means that all positive responses between ‘a little’ and ‘extremely’ were recoded and counted as ‘1’ while ‘not at all’ remained ‘0’. There were no negative worded questions hence no need for recoding. Thresholds for case identification in both sub-scales were determined by the use of standardized theoretical optimal cut-off points regarding sensitivity and specificity 13. A sum score of 5 or more in the somatoform subscale (SCL-SOM) depicts a somatoform disorder diagnosis with a sensitivity and specificity of 83/56.^13^

Prevalence of somatoform was determined using descriptive statistics. Pearson's Chi-Square was used to test bivariate associations in the disorders across demographic characteristics. Multivariate logistic regression analysis was used to estimate the odds of developing the outcome with displacement-related factors while controlling for statistically significant demographic correlates. Adjusted Odds Ratios (AORs) were determined with a 95% confidence interval (CI). Other predictors of the outcomes were selected a priori based on data from relevant literature and our theoretical assumptions. Only factors that were associated with the outcome of interest in the bivariate analysis were included in the respective multivariate analyses to eliminate the error of over-adjusting without compromising identification of the predictors for the outcomes of interest. The significance threshold was set at α = 0.05.

## Results

We gathered data from 335 respondents amounting to 85% response rate. Table 1 presents demographic data of respondents. Over half of the respondents (51%) were females. The mean age of the respondents in this present study was 34 ± 11. Respondents' ages ranged between 18 and 74 years. Majority of the respondents (63.3%) were young adults while older adults were the least number of respondents (4.8%). Most respondents were married (43.3%). Most of the respondents had attained secondary education (36.4%). Respondents were predominantly farmers. In terms of income, most respondents (57.9%) earned below minimum wage.

**Table 1:** Demographics of the study respondents

Variables	Frequency (335)	Percentage (%)
**Gender**		
Male	164	49
Female	171	51
**Age (in years)**		
18-35	212	63.3
36-55	107	31.9
>55	16	4.8
**Marital Status**		
Single	87	26
Married	145	43.3
Divorced	22	6.6
Separated	50	14.9
Widowed	31	9.3
**Educational attainment**		
No formal education	68	20.3
Primary	83	24.8
Secondary	122	36.4
Tertiary	62	18.5
**Occupation**		
Unemployed	75	22.4
Public Servant	26	7.8
Business Owner	82	24.5
Farmer	152	45.4
**Household size**		
1-3	202	60.3
4 and greater	133	39.7
**Household income per month**	78	23.3
No income earned	198	57.9
Below Minimum wage	46	13.7
Within minimum wage	9	2.7
Above minimum wage	8	2.4
I'd rather not say		
**Duration of displacement**		
About 1 month	22	6.6
> 1 month- < 6 months	91	27.2
> 6 months to 12 months	162	48.4
> 12 months	60	17.9

Table 2 showed that the proportion of respondents who reported developing somatoform disorder differed by marriage (χ^2^= 10.8 df= 4, *p*= 0.030). There was a significant association between level of education and somatoform disorder among the study respondents (χ^2^ = 9.38, df= 3, *p*= 0.030). There was a significant association between the occupation of respondents and somatoform disorder, χ^2^ =13.15 df= 3, *p*= 0.004. There was also a significant relationship between respondents' income and somatoform disorder, χ^2^=29.1, df =4, *p* <0.001. Furthermore, there was a significant relationship between duration of displacement and somatoform disorder, χ^2^ =24.9, df= 3, *p* < 0.001.

**Table 2:** Bivariate analysis of relationship between demographic factors with SSD

Variables	Presence of somatoform (N, %)	Absence of somatoform (N, %)	p-Value
**Gender**			
Male	94 (47.5)	67 (48.9)	0.521
Female	104 (52.5)	70 (51.1)	
**Age (in years)**			
18-35	119 (60.1)	93 (67.9)	0.231
36-55	67 (33.8)	40 (29.2)	
>55	12 (6.1)	4 (2.9)	
**Marital Status**			
Single	42 (21.2)	45 (32.8)	
Married	99 (50)	46 (33.6)	0.030*
Divorced	11 (5.6)	11 (8.0)	
Separated	30 (15.2)	20 (14.6)	
Widowed	31 (8.1)	15 (10.9)	
**Educational attainment**			
No formal education	33 (16.7)	35 (25.5)	
Primary	49 (24.7)	34 (24.8)	
Secondary	84 (42.4)	38 (27.7)	0.030*
Tertiary	32 (16.2)	30 (21.9)	
**Occupation**			
Unemployed	31 (15.7)	44 (32.1)	
Public Servant	17 (8.6)	9 (6.6)	
Business Owner	50 (25.3)	32 (23.4)	
Farmer	100 (50.4)	52 (38.0)	0.004*
**Household size**			
1-3	126 (63.6)	76 (55.5)	
4 and above	72 (36.4)	61 (44.5)	0.083
**Household income per month**			
No income earned	35 (17.7)	43 (31.4)	0.001**
Below Minimum wage	129 (65.2)	65 (47.4)	
Within minimum wage	25 (12.6)	21 (15.3)	
Above minimum wage	9 (4.5)	0 (0.0)	
I'd rather not say	0 (0)	8 (5.8)	
**Duration of displacement**			
About 1 month	7 (3.5)	15 (10.9)	
> 1 month- < 6 months	40 (20.2)	51 (37.2)	
> 6 months to 12	115 (58.1)	47 (34.3)	0.001**
months	36 (18.2)	24 (17.5)	
> 12 months			

* **Statistical significance based on *p*-Value < 0.05**

** **Statistical significance based on *p*-Value < 0.001**

As can be seen in Table 3, displacement-related factors significantly associated with somatoform disorder in the study population include discrimination as a result of present status, χ^2^ = 16.6, df= 1, *p*< 0.001; outbreak of various diseases including COVID-19, χ^2^ = 7.89, df= 1, *p* = 0.01; loss of loved ones due to crises or disasters, χ^2^= 18.3, df= 1, *p*< 0.001; living in overcrowded shelters, χ^2^ = 9.54, df= 1, *p*= 0.002; fears over reprisal attacks or natural disasters, χ^2^ = 15.5, df=1, *p* < 0.001; lack of basic amenities, χ^2^ = 8.56, df= 1, *p*= 0.003; and concerns about health or safety, χ^2^= 7.1, df= 1, *p*= 0.010.

**Table 3:** Bivariate analysis of displacement-related factors associated with SSD

Items	SSD		

	Yes N (%)	No N (%)	*p*-Value
Stress at work	142 (71.7)	56 (28.3)	0.250
Discrimination	50 (25.3)	148 (74.7)	<0.001**
Financial strain	190 (96.0)	8 (4.0)	0.070
Loss of loved ones in crises or natural disaster	73 (46.6)	125 (63.1)	<0.001**
Outbreak of diseases including COVID-19	187 (94.4)	11 (5.6)	0.010*
Overcrowded shelter	76 (38.4)	122 (61.6)	0.002*
Fears over reprisal attacks	67 (33.8)	131 (66.2)	<0.001**
Separated from family due to conflicts and/ or disasters	85 (43.4)	111 (56.6)	0.060
Insufficient amenities	180 (91.8)	16 (8.2)	0.003*
Domestic violence	52 (26.3)	146 (73.7)	0.650
Concerns about safety and general health	190 (96.0)	8 (4.0)	0.010*

* **Statistical significance based on *p*-Value < 0.05**

** **Statistical significance based on *p*-Value < 0.001**

Table 4 shows the multivariable logistic regression analysis of the Sociodemographic correlates and other factors associated with the somatoform disorder after adjusting for all potential covariates that were found significant in the chi-square analysis. However, regardless of their significance status for the outcome variable, age and gender were included in all the multivariable models due to their biological relevance.

**Table 4:** Multivariate analysis of sociodemographic correlates, displacement-related factors, and somatoform disorder

Variables	AOR	95% CI	*P*- Value
**Gender**			
Female	1	-	-
Male	0.71	0.39, 1.27	0.251
**Age (in years)**			
18-35	1	-	-
36-55	1.11	0.59, 2.07	0.741
>55	3.29	0.79, 13.6	0.101
**Marital Status**			
Single	1	-	
Married	2.80	1.17, 6.73	0.020*
Divorced	1.06	0.29, 3.78	0.091
Separated	1.51	0.54, 4.21	0.431
Widowed	1.25	0.39, 4.04	0.711
**Level of Education**			
No formal education	1	-	-
Primary	1.24	0.55, 2.81	0.611
Secondary	1.48	0.68, 3.19	0.321
Tertiary	0.54	0.19, 1.53	0.251
**Occupation**			
Unemployed	1	-	-
Public Servant	1.96	0.37, 10.8	0.433
Business Owner	1.74	0.62, 4.84	0.293
Farmer	1.18	0.48, 2.98	0.701
**Household Monthly Income**			
No income earned	1	-	-
Below Minimum wage	0.69	0.28, 1.68	0.421
Within minimum wage	0.37	0.12, 1.16	0.091
Above minimum wage	250	0.001	0.990
I'd rather not say	-	-	-
**Duration of displacement**			
About 1 month	1	-	-
> 1 month- < 6 months	1.01	0.28, 3.63	0.985
> 6 months to 12 months	3.29	0.95, 11.3	0.003*
> 12 months	1.51	0.39, 5.92	0.560
**Discriminated because of present status**			
No	1	-	-
Yes	2.25	1.21, 4.17	0.010*
**Loss of loved ones due to crises or natural disasters**			
No	1	-	-
Yes	1.34	0.42, 4.32	0.030*
**Outbreak of diseases including the COVID-19 affected you**			
No	1	-	-
Yes	1.92	1.07, 3.45	0.030*
**Overcrowded shelter**			
No	1	-	-
Yes	2.30	1.25, 4.26	0.008*
**Fears over reprisal attacks or natural disasters**			
No	1	-	-
Yes	2.05	1.09, 3.86	0.030*
**Concerned about health and safety**			
No	1	-	-
Yes	1.29	0.41, 4.06	0.660

* **Statistical significance based on *p*-Value < .05; AOR= Adjusted Odds Ratio; CI= Confidence Interval (95%)**

Being married was a significant correlate of somatoform disorder in the study population, b = 1.26, *p* = 0.020, AOR =2.80 (95% CI: 1.17, 6.73). This implies that displaced persons who are married were almost 3 times more likely than people in other marital groups to develop the somatoform disorder. The duration of displacement was also a significant correlate of somatoform disorder in the study population, b = 1.19, *p* = 0.003, AOR =3.29 (95% CI: .954, 11.3). In other words, persons displaced for between 6 months and 1 year are almost 4 times more likely to develop the somatoform disorder than those who may have been displaced for shorter periods. Discrimination because of present status showed a significant relationship with development of somatoform disorder, b = 0.81, *p* = 0.010, AOR =2.25 (95% CI: 1.21, 4.17). Hence, those who were discriminated against because of their displacement status were 2.25 times more likely than those not discriminated against to develop the somatoform disorder in the study population. Being affected by the outbreak of diseases including the novel Coronavirus 2019 (COVID-19) showed a significant relationship with the development of the somatoform disorder in the study population, b = 0.29, *p* = 0.030, AOR =1.92 (95% CI: 1.07, 3.45). Furthermore, we found a significant relationship between loss of loved ones due to crises or disasters and development of the somatoform disorder in the study population, b = 0.65, *p* = 0.030, AOR =1.92 (95% CI: 1.07, 3.45). This implies that displaced persons who suffered the loss of loved ones due to crises or disasters were almost two times more likely than those who did not suffer losses to develop the somatoform disorder. There was also a significant relationship between overcrowded shelters and developing the somatoform disorder in the study population, b = 0.84, *p* = 0.008, AOR = 2.30 (95% CI: 1.25, 5.26), implying that those who reported poor living conditions such as overcrowding were more than 2 times more likely than others who had better living conditions to suffer from the somatoform disorder. Fear over reprisal attacks or reoccurrence of natural disasters showed a significant relationship with developing the somatoform disorder in the study population, b = 0.72, p= 0.030, AOR=2.05 (95% CI: 1.09, 3.86), implying that displaced persons who had fear of reprisals and reoccurrence were over 2 times more likely to develop the somatoform disorder.

## Discussion

Overall, the results of this present study showed a high prevalence of the outcomes in the study area. Although there is a paucity of data on identified somatic symptoms among displaced persons in the African region, a Ugandan study 14 found that the prevalence of somatic symptoms among war-affected displaced youths in Northern Uganda was 28.4%. Another East-African study 15 also found the prevalence of somatic discomforts to be 43.8% among Somalis displaced in Finland. All of these figures are moderately lower than the prevalence found in this present study (59.1%). These disparities in prevalence across the different studies may be due to the differences in methodology as well as the standardized instruments used. Molsa et al 15 used the General Health Questionnaire (GHQ-12), and Amone-P'Olak and Omech 14 used the African Youth Psychosocial Assessment Instrument (APAI). Our study gives results in terms of the presence or absence of the condition according to the core symptoms defined in the WHO-International Classification of Disease-10 Revision. Disparities in prevalence across geographical lines may also be a result of higher levels of trauma in the different home countries at different periods.

Being married was significantly associated with being symptomatic to somatoform. Though not significantly associated with somatoform in the bivariate analysis, domestic violence which occurred more frequently in females in this study could be a possible reason for this finding. This is consistent with a Ugandan study 14 which found being married as a significant predictor of somatoform disorder. Furthermore, prolonged displacement was also and significantly associated with somatoform in the study population. Persons displaced for longer durations were almost 4 times more likely than persons displaced for shorter periods to develop the outcome. This is consistent with a Sri Lankan study that found a significant relationship between prolonged displacement and somatization.^16^ This is also consistent with two studies. 14, 15 A possible reason for this may be that displacement for long periods leads to exposure to many vices such as poor host community acceptance and hence further attacks; frustration leading to domestic violence; economic crises due to increasing population; inflation, etc.

Discrimination, being affected by the outbreak of diseases including the COVID-19, loss of loved ones due to conflicts and/ or disasters, overcrowded shelters, and fear of reprisal attacks or natural disasters were factors most significantly associated with being symptomized to somatoform disorder in the study population. A study 14 also found that harm to loved ones was significantly associated with being symptomized to somatoform among displaced persons in Northern Uganda. This present study also agrees with findings 14 that identified an outbreak of other disease stressors, stigma, discrimination, and poor community relations to be significantly associated with somatoform symptoms. A study 17 cited that displaced persons were more likely to be affected by the COVID-19 because of some of their overcrowded shelters, barriers to access to social services and health care, and financial insecurity. Fear of reprisal attacks may be an associated factor of somatoform symptoms because due to this, people continue to migrate without any permanent shelter. In the course of migration, they may be exposed to psychological stressors that may impact on their physical body. Some may encounter more attacks as a result of their migration status.

## Conclusion

This study highlights the prevalence and correlates of somatoform disorder among IDPs in Ogoja LGA, Nigeria. The findings suggest that the high prevalence of the outcome studied may be related to many life stressors and events. In other words, the findings suggest rising rates of mental health problems consistent with those from previous literature globally.

## Implications

These findings suggest that an increase in the coverage and availability of mental health services in the forgotten IDPs of Ogoja should be prioritized while making efforts to minimize people's repeated exposure to traumatic events. Clinical interventions should focus on providing access to mental health services and community mental health promotion innovations to improve IDPs' coping strategies and treatment opportunities. Persons identified to be positive to SSD should be targeted in policy formulation processes and in the planning of culturally-sensitive interventions

## Limitations

This is a cross-sectional study hence some findings should be read with caution. Secondly, factors studied in this present study were self-reported and may be affected by recall bias. Longitudinal studies on evidence-based interventions for mental health will help to address these factors.
